# Predictive role of renal resistive index for clinical outcome after revascularization in hypertensive patients with atherosclerotic renal artery stenosis: a monocentric observational study

**DOI:** 10.1186/1476-7120-12-9

**Published:** 2014-02-20

**Authors:** Rosa Maria Bruno, Elena Daghini, Daniele Versari, Melania Sgrò, Michela Sanna, Luigi Venturini, Caterina Romanini, Irene Di Paco, Isabella Sudano, Roberto Cioni, Lilach O Lerman, Lorenzo Ghiadoni, Stefano Taddei, Stefania Pinto

**Affiliations:** 1 Department of Clinical and Experimental Medicine, University of Pisa, Pisa, Italy; 2 Institute of Clinical Physiology – CNR, Via Moruzzi 1, 56124 Pisa, Italy; 3 University Heart Center, University Hospital Zurich, Zurich, Switzerland; 4 Department of Interventional Radiology, University Hospital of Pisa, Pisa, Italy; 5 Department of Internal Medicine, Division of Nephrology and Hypertension, Mayo Clinic College of Medicine, Rochester, MN USA

**Keywords:** Resistive index, Ultrasound, Renal artery stenosis, Hypertension, Revascularization

## Abstract

**Background:**

The present study evaluated the predictive value of renal resistive index (RI) for renal function and blood pressure (BP) outcome in hypertensive patients with unilateral atherosclerotic renal artery stenosis submitted to successful revascularization.

**Methods:**

In 158 hypertensive patients with atherosclerotic renal artery stenosis RI was acquired. Twelve months after revascularization, they were classified on the basis of renal function and BP outcome as benefit (BP < 140/90 mmHg or diastolic BP reduction > 15 mmHg with the same of reduced drugs; decrease in glomerular filtration rate > 20%), or failure.

**Results:**

Regarding renal function outcome, RI in the stenotic and in the contralateral kidney were significantly higher in patients with failure (n = 20) than in those with benefit (0.72 ± 0.11 vs 0.61 ± 0.11 and 0.76 ± 0.08 vs 0.66 ± 0.09, p < 0.05). Among different cutpoints generated, RI in the contralateral kidney >0.73 provided the largest area under the curve (0.77), and the highest sensitivity (80%) and specificity (72%). In the multivariate logistic regression analysis, RI in the contralateral kidney >0.73 was an independent predictor of a failure in renal function outcome.

Regarding BP outcome, patients with no benefit from revascularization (n = 60) had similar RI in the stenotic and contralateral kidney (p = ns), but presented higher pulse pressure, albuminuria and hypertension duration in comparison to patients with improved BP control.

**Conclusions:**

RI in the contralateral kidney is an independent predictor of renal function outcome after successful revascularization in hypertensive patients with unilateral atherosclerotic renal artery stenosis, whereas it is not able to predict blood pressure outcome.

## Background

In the last decades the prevalence of atherosclerotic renal artery stenosis (ARAS), possible cause of secondary hypertension and ischemic nephropathy, has progressively increased in Western countries, due both to the aging of the population and to improved technical capability of detecting it [1]. The debate of treating ARAS by using medical therapy or interventional procedures, such as angioplasty with or without stenting, is still open [2-7]. Considering the large variability among patients in terms of risk factors and global risk, as well as the stage of renal damage, the revascularization procedure in an unselected population might be associated with variable outcome. Therefore, at the moment the real challenge is the possibility to identify reliable parameters that can be used to detect patients that could benefit from such procedure.

The renal resistive index (RI), measured by duplex ultrasound, has been suggested to be a useful prognostic tool, that enables to identify patients who will not benefit from revascularization in terms of renal function or blood pressure (BP) improvement [8-11]. In particular Radermacher and coauthors showed that a cut-off of 0.80 presented an excellent sensitivity (96%), despite a low specificity (53%) [9]. After the brilliant report of this single-center study, other authors tested the predictive role of RI for renal function and BP outcome, with conflicting results [12]. Indeed, some studies confirmed a predictive role of increased RI for either renal function [13,14] and BP outcome [13,15-17], while in other studies the association of increased RI and renal [8,15] or BP outcome [18] was inconsistent. Furthermore, these studies are heterogeneous regarding chosen cut-off, definition of outcome, clinical characteristics of the studied population, duration of follow-up, type of intervention, and sample size, making difficult to draw clear conclusions.

Therefore, the aim of the present study was to evaluate the prognostic value of baseline RI in the stenotic and controlateral kidney in predicting the outcome (12-month follow up) of BP and renal function in hypertensive patients with unilateral ARAS submitted to successful revascularization.

## Methods

### Patients

One hundred and sixty eight patients (mean age 61.2 ± 11.3 years, male 64.9%) were enrolled from 1990 to 2008. Inclusion criteria were:

Unilateral atherosclerotic renal artery stenosis >60% defined by a renal to aortic ratio greater than 3.5 at duplex ultrasound examination [19], confirmed by angio-magnetic resonance or spiral computed tomography as recommended [20];

Diagnosis of arterial hypertension according to current Guidelines [21], with or without chronic kidney disease;

Patient’s informed consent;

Exclusion criteria were: fibromuscolar dysplasia; bilateral renal artery stenosis; age > 80 years; KDOQI stage 5 chronic kidney disease (glomerular filtration rate <15 ml/min or dialysis); history of severe adverse reaction to iodinated contrast; technical limitations to revascularization procedure; severe comorbidities that contraindicated the intervention according to clinical judgment.

The study was approved by the ethical committee of the Azienda Ospedaliero-Universitaria Pisana and conformed the Declaration of Helsinki. In accordance with institutional guidelines, all patients were aware of the investigational nature of the study and gave written consent to it.

### Study design

All the patients underwent abdominal duplex ultrasonography (Technos and MyLab25, Esaote, Florence, Italy) in the month before revascularization after fasting 6–8 hours. The study was performed by the same operators (R.M.B and M.S.) using a high resolution multifrequency (2.5 - 4.5 MHz) Convex probe. Three velocimetric measurements of the interlobar renal arteries adjacent to medullary pyramids were obtained by a translumbar and/or anterior approach and RI was calculated according to the formula: RI = (systolic peak velocity – end diastolic velocity)/systolic peak velocity, as already reported in previous studies [22].

Office BP (mean of at least two BP values measured after 5 minute sitting position by mercury sphygmomanometer) was measured by a trained physician. Office BP was measured in both arms at the first visit in our center and then measured in subsequent visits in the arm in which resulted to be higher. Blood samples were collected in all patients to measure serum creatinine and other routine parameters, by standard technique. Glomerular filtration rate was estimated using the Modification of Diet in Renal Disease (MDRD) formula [23].

Angioplasty was performed following Tegtmeyer technique [24], by using balloon catheters with or without stent placement following the technique described by Rees [25]. Digital angiography was performed after the revascularization to verify the immediate technical success, defined as the absence of stenosis or residual stenosis < 30%, without complications. Vessel patency was checked after 24 hours, and after 1-3-6-12 months using duplex ultrasonography, and confirmed if necessary with MRI or CT angiography, and subsequent digital angiography, defining restenosis as a reduction ≥50% in angiographic diameter. In particular, angiography was repeated if, according to clinical judgment, a new revascularization procedure was recommended. A clinical follow-up, consisting in physical examination, office BP measurement, routine blood exams including serum creatinine, was performed after 1-3-6-12 months. BP and renal outcome were defined according to the American Heart Association guidelines for the reporting of renal artery revascularization in clinical trials [26]. Twelve months after revascularization patients were classified according to BP outcome as:

•“Benefit” if: diastolic blood pressure < 90 mm Hg and/or systolic blood pressure < 140 mm Hg without pharmacological treatment or on the same or reduced number of medications (or reduced number of defined daily doses); reduction in diastolic blood pressure by at least 15 mm Hg on the same or reduced number of medications;

•“Failure” if the abovementioned criteria were not met [26].

Twelve months after revascularization, patients were also classified in two categories, according to renal outcome:

•“benefit” if there was an increase in eGFR ≥20% compared to pretreatment values or a value of eGRF within ±20% of pretreatment values;

•“failure” if there was a deterioration in eGFR ≥20% after treatment [6,26].

### Data analysis

Statistical analysis was performed by means of NCSS 2004 software (NCSS, Kaysville, Utah). Results are expressed as mean ± SD. Differences between two means were compared by the Student’s t test for paired or unpaired observations, as appropriate, while categorical variables were compared using the chi-square test. Differences were considered to be statistically significant when p was <0.05.

Receiver operating characteristic (ROC) analysis was performed to calculate the best threshold value of RI associated with a benefit of revascularization in terms of BP and renal function outcome, both defined as dichotomous variables as explained above. Cut-points selected were those that yielded the greatest sum of sensitivity and specificity. Logistic regression analysis was used to identify independent predictors of clinical outcome, according to clinical and ultrasonographic parameters.

## Results

### Baseline characteristics

Baseline characteristics of the study population are summarized in Table 1. At baseline, RI was significantly lower in the stenotic kidney as compared to the contralateral, non stenotic, kidney, regardless of the side affected (0.62 ± 0.12 vs 0.67 ± 0.09, p < 0.0001). The percentage of nephrosclerosis, evaluated as RI >0.70 [27] in the contralateral kidney, was 39%. Reno-aortic ratio was 4.1 ± 0.9 in the stenotic kidney and 1.7 ± 0.8 in the contralateral kidney (p < 0.0001). According to renal function at baseline, 15 patients were classified in KDOQI (Kidney Disease Outcomes Quality Initiative) stage 1, 70 in stage 2, 72 in stage 3, 11 in stage 4.

**Table 1:** Clinical and ultrasonographic parameters of the study population at baseline

	**Overall population (n = 168)**	
**Clinical parameters**		
**Age (years)**	61.2 ± 11.3	
**Male sex (%)**	64.9	
**BMI (Kg/m**^**2**^**)**	25.4 ± 3.8	
**Smoking (%)**	30.2	
**Previous cardiovascular events (%)**	25.9	
**Hypercholesterolemia (%)**	73.1	
**Diabetes (%)**	16.7	
**Hypertension duration (years)**	8.6 ± 8.5	
**Systolic blood pressure (mmHg)**	161.6 ± 21.2	
**Diastolic blood pressure (mmHg)**	90.0 ± 13.8	
**Pulse pressure (mm Hg)**	71.7 ± 16.9	
**Number of antihypertensive drugs**	2.1 ± 0.9	
**RAS-blockers therapy**	60.0	
**Antithrombotic therapy (%)**	60.5	
**Cholesterol-lowering therapy (%)**	37.5	
**Serum creatinine (mg/dl)**	1.33 ± 0.61	
**Creatinine clearance (ml/min)**	67.2 ± 28.9	
**Estimated GFR (ml/min/1.73 m**^**2**^**)**	61.1 ± 20.5	
**Albuminuria (mg/24 h)**	98.9 ± 278.8	
**Ultrasonographic parameters**	**Stenotic kidney**	**Contralateral kidney**
**Resistive index**	0.62 ± 0.12*	0.67 ± 0.09
**Pulsatility index**	1.09 ± 0.36*	1.22 ± 0.31
**Renal longitudinal diameter**	10.5 ± 1.3	10.7 ± 1.5
**Renal to aortic ratio**	4.1 ± 0.9*	1.7 ± 0.8

*p < 0.05 vs contralateral kidney.

All the patients underwent angioplasty with (n = 104) or without stent (n = 64). Before 1995, angioplasty without stenting was the only kind of intervention performed (n = 25); from 1995 to 2003, angioplasty with stent placement was performed mainly in ostial stenosis (n = 51 out of 85); from 2003 to 2008, stent placement was performed in all but 5 cases in which was technically not feasible. The rate of procedure-related major clinical adverse events was 2.4% (3 cases requiring total nephrectomy; 1 arterial thrombosis treated with endoarterectomy), whereas a residual stenosis was demonstrated soon after the revascularization procedure in 1 patient. Two cases of retroperitoneal bleeding/hematoma and 3 cases of partial renal ischemia, not requiring medical or surgical intervention and without any clinical sequelae, also occurred. During 12-month follow-up, 24 restenosis in the site of the intervention occurred, 9 patients developed contralateral renal artery stenosis, 1 patient died for a cardiovascular event and 1 patient started dialysis. In total 10 patients were excluded (4 major procedure-related adverse events, 1 death, 1 dialysis, 4 lost to follow-up), thus the following analysis were performed on 158 patients.

### Effect of type of intervention

Clinical and ultrasonographic characteristics, BP and renal outcome and restenosis rate were analyzed according to the type of intervention: percutaneous angioplasty with stent placement (n = 97) versus angioplasty only (n = 61). At baseline the two groups resulted to be substantially similar for clinical and ultrasonographic characteristics, except for a greater diastolic BP in the angioplasty-only group and a greater use of antithrombotic and lipid-lowering drugs in the stenting group, the latter probably due to change in guidelines recommendations over years (data not shown). No significant difference in terms of restenosis rate (15 vs 9 cases, p = 0.87) and BP outcome failure (65 vs 34 cases, p = 0.18) was encountered between patients undergoing angioplasty with or without stenting. Renal outcome failure tended to be more frequent in subjects receiving only angioplasty (8 vs 12 cases, p = 0.07).

### Renal function outcome

After 12-month follow-up, a failure in renal function outcome was demonstrated in 20 patients, while in 138 patients glomerular filtration rate was unchanged (n = 101) or improved (n = 36). Patients with worsened renal function were older, were more frequently males, had a higher body mass index, higher systolic and pulse pressure values in comparison to the group who showed benefit from revascularization (Table 2). At baseline, higher serum creatinine and proteinuria and a lower glomerular filtration rate (Table 2) were demonstrated in the failure group: in particular, 6 out of 11 patients in KDOQI class 4 experienced an eGFR decline greater than 20%. As ultrasonographic parameters are concerned, RI in the stenotic and in the contralateral kidney were both significantly higher in the failure than in the benefit group (p = 0.001 for both, Table 2), while the RI difference between the two kidneys was not significantly different (0.03 ± 0.09 vs 0.06 ± 0.10, p = ns).

**Table 2:** Clinical and ultrasonographic parameters of the study population at baseline according to renal function outcome

	**Benefit (n = 138)**		**Failure (n = 20)**	
**Clinical parameters**				
**Age (years)**	60.3 ± 10.3†		67.8 ± 10.1	
**Male sex (%)**	63.5†		88.9	
**BMI (Kg/m**^**2**^**)**	25.2 ± 3.6†		27.5 ± 4.1	
**Current smoking (%)**	32.2		18.8	
**Previous cardiovascular events (%)**	28.7		37.5	
**Hypercholesterolemia (%)**	74.8		68.4	
**Diabetes (%)**	14.4		18.8	
**Hypertension duration (years)**	9.1 ± 8.5		6.1 ± 4.9	
**Systolic blood pressure (mmHg)**	161.7 ± 21.2†		172.2 ± 21.0	
**Diastolic blood pressure (mmHg)**	91.5 ± 13.9		88.2 ± 13.8	
**Pulse pressure (mm Hg)**	70.5 ± 16.5†		84.0 ± 14.9	
**Number of antihypertensive drugs**	2.1 ± 1.0		2.1 ± 1.0	
**RAS-blockers therapy**	72.2		100.0	
**Antithrombotic therapy (%)**	60.4		58.3	
**Cholesterol-lowering therapy (%)**	35.2		31.6	
**Serum creatinine (mg/dl)**	1.24 ± 0.42†		2.06 ± 1.19	
**Creatinine clearance (ml/min)**	68.2 ± 25.6†		50.2 ± 39.2	
**Estimated GFR (ml/min/1.73 m**^**2**^**)**	63.3 ± 18.9†		45.3 ± 24.1	
**Albuminuria (mg/24 h)**	90.8 ± 282.8		295.3 ± 464.5	
**Ultrasonographic parameters**	**Stenotic kidney**	**Contralateral kidney**	**Stenotic kidney**	**Contralateral kidney**
**Resistive index**	0.61 ± 0.11*†	0.66 ± 0.09†	0.72 ± 0.11*	0.76 ± 0.08
**Pulsatility index**	1.04 ± 0.31*†	1.19 ± 0.29†	1.36 ± 0.37*	1.45 ± 0.34
**Renal longitudinal diameter**	10.7 ± 1.3	10.8 ± 1.6	10.4 ± 1.3	10.2 ± 1.7
**Renal to aortic ratio**	4.3 ± 1.0*	1.9 ± 1.1	3.8 ± 0.8*	1.4 ± 0.9

*p < 0.05 vs contralateral kidney, †p < 0.05 vs failure group.

**Figure 1:**
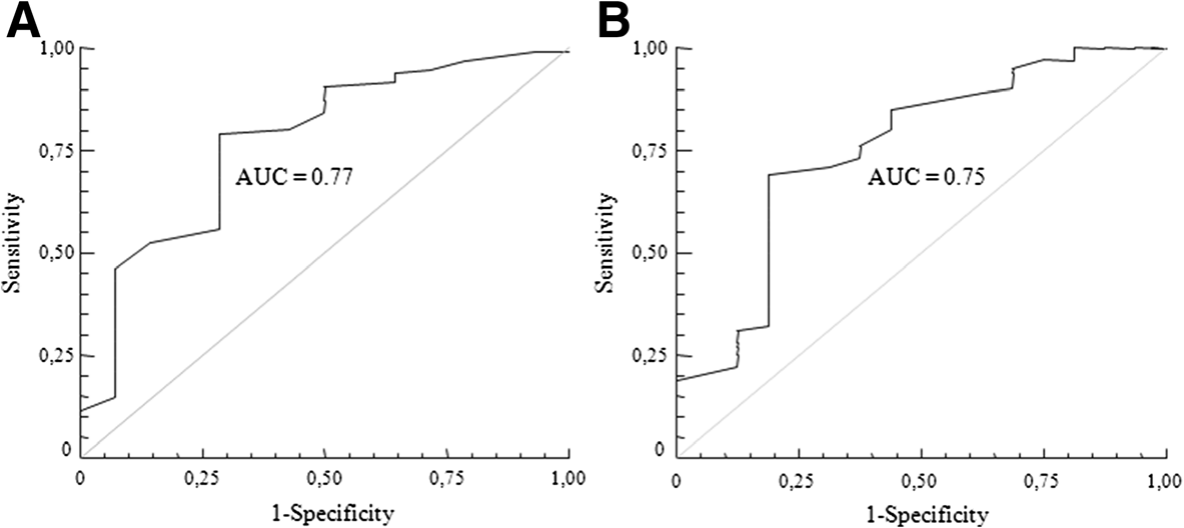
Receiver-operating characteristic curves of resistive index in the contralateral (A) and in the stenotic (B) kidney for renal function worsening.

ROC analysis of main ultrasonographic and clinical parameters was performed and showed in Table 3. RI in the contralateral kidney had the largest AUC among the parameters considered, indicating that it was the most suitable in predicting benefit in terms of renal function outcome after revascularization. Among different cutpoints generated, RI in the contralateral kidney >0.73 provided the highest sum of sensitivity and specificity (Table 3, Figure 1).

**Table 3:** Receiver-operating characteristic analysis of the optimal cut-points of parameters in predicting renal function worsening after revascularization

**Parameters**	**AUC**	**95% confidence interval**	**Cutpoint**	**Sensitivity (%)**	**Specificity (%)**
**RI contralateral kidney**	0.77	0.59-0.88	0.73	80	72
**RI stenotic kidney**	0.75	0.57-0.86	0.67	81	69
**RI difference**	0.58	0.38-0.70	0.09	79	34
**PI contralateral kidney**	0.73	0.55-0.84	1.32	69	75
**PI stenotic kidney**	0.74	0.54-0.85	1.18	80	68
**Estimated GFR**	0.73	0.55-0.85	48 ml/min 1.73 m^2^	79	67
**Serum creatinine**	0.74	0.55-0.86	1.8 mg/dl	59	91
**Pulse pressure**	0.72	0.60-0.82	75 mmHg	61	73

**Figure 2:**
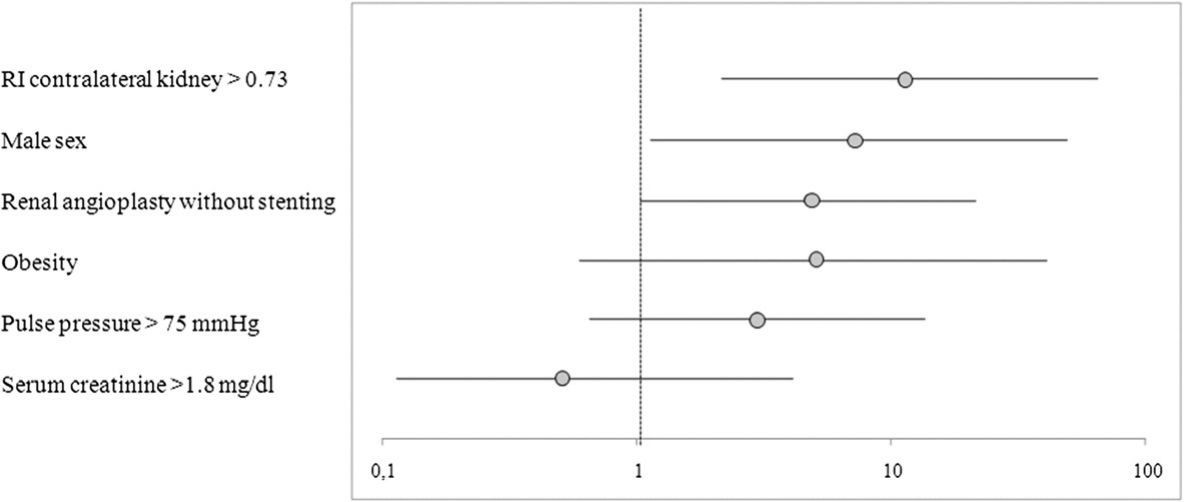
**Odds ratio (95% confidence interval) for renal function worsening after revascularization in a multivariate logistic regression analysis.** The model comprised also age as a continuous variable.

In order to verify whether the suggested cut-off was associated to a unfavourable renal outcome independently of confounding factors, and particularly of baseline renal function, a multivariate logistic regression analysis was performed. In a model comprising also age, gender, obesity, pulse pressure > 75 mmHg, serum creatinine > 1.80 mg/dl, type of intervention performed, RI in the contralateral kidney > 0.73 remained an independent predictor of a failure in renal function outcome (OR 11.3: CI 95% 1.9-66.1) (Figure 2).

At one year follow up, clinical characteristics of patients classified according renal function outcome were also analyzed. Patients in the failure group presented a significantly higher serum creatinine (3.30 ± 2.03 vs 1.14 ± 0.35 mg/dl, p < 0.05) and lower glomerular filtration rate (27.6 ± 21.9 vs 66.7 ± 26.9 ml/min, p < 0.05) in comparison to their counterparts. Despite similar systolic and diastolic BP were observed (148.8 ± 14.3/76.8 ± 11.3 vs 142.7 ± 17.1/81.9 ± 10.9 mmHg, p = ns), pulse pressure (71.9 ± 13.3 vs 60.7 ± 15.2 mmHg, p < 0.05) and the number of hypertensive drugs (2.5 + 1.2 vs 1.6 + 1.3, p < 0.05) were significantly greater in the failure group.

### BP outcome

On the basis of BP outcome, 98 patients were classified in the benefit category and 60 in the failure category. Since a higher restenosis rate was encountered in the failure group (18 vs 6 cases, p < 0.0001), statistical analysis was performed with the exclusion of the patients who experienced restenosis (Table 4). Among clinical baseline characteristics, the benefit group had higher diastolic and lower pulse pressure values, shorter hypertension duration, and milder proteinuria. On the contrary, patients with positive or negative BP outcome were not different for any of the considered ultrasonographic parameters (Table 4). In particular RI between the benefit and the failure group was similar, both in the stenotic (0.62 ± 0.13 vs 0.65 ± 0.12, p = ns) and in the controlateral (0.67 ± 0.10 vs 0.68 ± 0.11, p = ns) kidney, as well as RI difference between stenotic and contralateral kidney (0.06 ± 0.11 vs 0.04 ± 0.10, p = ns). Results were superimposable when patients who experienced restenosis were included (data not shown).

**Table 4:** Clinical and ultrasonographic parameters of the study population at baseline according to blood pressure outcome

	**Benefit (n = 90)**		**Failure (n = 42)**	
**Clinical parameters**				
**Age (years)**	62.0 ± 10.1		61.3 ± 12.1	
**Male sex (%)**	64.4		61.9	
**BMI (Kg/m**^**2**^**)**	25.7 ± 3.6		25.0 ± 3.5	
**Current smoking (%)**	28.2		33.3	
**Previous cardiovascular events (%)**	25.3		31.4	
**Hypercholesterolemia (%)**	77.5		64.1	
**Diabetes (%)**	18.3		17.1	
**Hypertension duration (years)**	7.3 ± 7.1†		12.1 ± 11.1	
**Systolic blood pressure (mmHg)**	162.6 ± 22.8		161.9 ± 17.8	
**Diastolic blood pressure (mmHg)**	92.5 ± 13.7 †		85.9 ± 14.2	
**Pulse pressure (mm Hg)**	70.1 ± 16.3†		76.3 ± 17.5	
**Number of antihypertensive drugs**	2.1 ± 0.9		2.2 ± 1.0	
**RAS-blockers therapy**	58.8		62.2	
**Antithrombotic therapy (%)**	61.9		58.9	
**Cholesterol-lowering therapy (%)**	36.7		31.0	
**Serum creatinine (mg/dl)**	1.37 ± 0.66		1.36 ± 0.69	
**Creatinine clearance (ml/min)**	64.3 ± 30.4		69.6 ± 27.6	
**Estimated GFR (ml/min/1.73 m**^**2**^**)**	59.9 ± 20.8		61.4 ± 22.4	
**Albuminuria (mg/24 h)**	22 ± 39†		359 ± 520	
**Ultrasonographic parameters**	**Stenotic kidney**	**Contralateral kidney**	**Stenotic kidney**	**Contralateral kidney**
**Resistive index**	0.62 ± 0.13*	0.67 ± 0.10	0.65 ± 0.12*	0.68 ± 0.11
**Pulsatility index**	1.07 ± 0.35*	1.21 ± 0.28	1.17 ± 0.39*	1.27 ± 0.42
**Renal longitudinal diameter**	10.5 ± 1.2	10.8 ± 1.7	10.4 ± 1.6	10.5 ± 1.6
**Renal to aortic ratio**	4.1 ± 0.9*	1.8 ± 1.0	3.9 ± 1.1*	1.7 ± 0.9

*p < 0.05 vs contralateral kidney, †p < 0.05 vs failure group.

## Discussion

The present study demonstrated that RI in the contralateral kidney is an independent predictor of renal function outcome after successful revascularization in hypertensive patients with unilateral atherosclerotic renal artery stenosis, whereas it is not able to predict blood pressure outcome.

Past and ongoing trials [2-6] have been trying to randomly compare revascularization procedures and medical therapy in populations of hypertensive patients with ARAS in order to establish whether interventional approach could be beneficial in terms of BP and renal function outcome. Recently, ASTRAL, STAR and CORAL trials [5-7] showed no difference in outcome in patients randomly assigned to either receive renal artery revascularization or medical treatment. These discouraging results strengthened the necessity for reliable selection criteria, not only to achieve successful results in the potentially curable patients but also to avoid adverse events related to the procedure itself in patients that would not benefit from the interventional approach [28,29].

Renal RI has been demonstrated to be useful as a negative prognostic index for renal function outcome by Radermacher and colleagues [9,10], showing that an RI >0.80 identifies patients who would not benefit from revascularization in terms of renal function. The ability to identify such patients is particularly important, because preservation of renal function is one of main rationales for performing angioplasty in patients with renal artery stenosis. However the cited paper left several questions open. First, this cut-off identifies a very small subset of aged patients with cardiovascular comorbidities, advanced atherosclerosis, chronic kidney failure, that might be probably excluded from interventional procedures: in our cohort, which is younger and with a lower prevalence of severe chronic kidney disease and previous cardiovascular events, only 19 out of 168 patients showed RI > 0.80 either in the stenotic or in the contralateral kidney. Our results suggest that in settings characterized by lower cardiovascular and renal morbidities, the cut-off of 0.80 is hardly applicable, thus explaining conflicting results found by other authors [8,13-18]. Second, it is not clear whether the 0.80 cut-off should refer to stenotic or controlateral kidney, given the profoundly different hemodynamic conditions to which the two kidneys are exposed. Contralateral kidney evaluation might be more correct from a pathophysiological point of view, since it is the one openly exposed to BP overload. However, the approach suggested in this paper is limited to unilateral atherosclerotic lesions.

The present study showed a 13% of patients who had worsened renal function, defined as a GFR decline of at least 20%, one year after revascularization. RI in the stenotic as well as in the contralateral kidney were both significantly higher in patients with no benefit in terms of renal function in comparison to their counterparts; this is in line with the previous report by Radermacher and coauthors, suggesting an high RI as a significant negative prognostic factor [9]. We considered different ultrasonographic parameters to identify the best predictor, and compared them to main clinical determinants of renal function outcome: baseline renal function and blood pressure. We found that RI in the contralateral kidney >0.73 provided the largest area under the curve, and acceptable values for sensitivity (80%) and specificity (72%). Furthermore, the logistic regression analysis indicated baseline RI in the contralateral kidney as an independent predictor of renal function outcome, even after adjustment for clinical characteristics that were associated with a worse outcome, suggesting that it could contribute to select those patients who will not benefit from renal revascularization. However, it should be taken into account that sensitivity and specificity were probably not sufficiently high to put this index right into clinical practice; moreover, the superiority in comparison to routine evaluation, such as serum creatinine at baseline, resulted to be limited.

It is interesting to note that in a relevant percentage of patients the contralateral kidney was characterized by nephrosclerosis, identified by a RI > 0.70 [27]. Indeed, the presence of stenosis leads to an activation of systemic mechanisms which can induce the progression of the atherosclerotic process and renal damage [30]. Theoretically, contralateral kidney is the one openly exposed to BP overload, without the paradoxical protection of the stenosis. Therefore, RI in the contralateral kidney can be considered the direct expression of the actual renal damage caused by high BP over time and by the consequent activation of local detrimental processes such as oxidative stress and inflammation, in parallel to what occurs in essential hypertension [31,32]. Worth of note, the greater sensitivity and specificity in predicting the renal function outcome has been reached when RI in the contralateral kidney rather than in the stenotic kidney has been analyzed.

The present study confirmed previous results [33] that showed a lower RI in the stenotic kidney as compared to the RI in the contralateral kidney, likely due to the post stenotic vasodilation in the affected kidney apparently compensating the presence of the stenosis. Accordingly, we tested RI difference between contralateral and stenotic kidney as a possible predictor of revascularization outcome, since it might represent an index of renal compensatory vasodilation, in the hypothesis that a higher RI difference could correspond to a less advanced renal damage in the stenotic kidney. Although RI difference tended to be higher in both the groups with BP and renal function benefit, these results did not reach statistical significance. Preliminary data from our group suggest that measuring RI difference after a vasodilatory stimulus might be an effective test to predict BP outcome [34].

In terms of BP outcome, the results of the present study, showing a 64% of cured/improved and a 32% of patients with no benefit at one year follow up after revascularization, are consistent with those of previous studies [2-4]. Our study failed to demonstrate a predictive role of any of the considered ultrasonographic parameters for BP outcome. On the other hand, hypertension duration, rather than age, appear to influence BP outcome after revascularization, conceivably by means of development of structural vascular changes, that are hardly reversible [35]. This hypothesis is supported by the fact that pulse pressure, a surrogate marker of large artery stiffness [36], and albuminuria, considered a marker of widespread vascular damage [37] in the hypertensive population, are among the parameters associated with worse BP outcome. Recently Leeser and collegues demonstrated that evaluation of translesional pressure gradients allows a satisfying BP improvement prediction [38]. Thus it is conceivable that also local factors such as the entity of the stenosis, play a major role in BP response to revascularization. Furthermore, the use of office BP instead of 24-hour BP monitoring, one of the limitations of the present study, could have contributed to lack of significance of the results regarding BP outcome.

### Perspectives

These data indicate that duplex ultrasound evaluation of RI in the contralateral kidney is an independent predictor of renal function outcome after successful revascularization in hypertensive patients with unilateral atherosclerotic renal artery stenosis, whereas it is not able to predict blood pressure outcome.

The results of the present study suggest that RI evaluation could contribute, at least in part, to better identify patients that could benefit from revascularization. At the moment, renal artery revascularization has not been demonstrated to be superior to medical therapy in hypertensive patients with RAS, possibly due to inadequate patient selection. Thus it is desirable to design further studies with the aim of identify non-invasive, highly predictive diagnostic tests in order to limit renal revascularization to patients who will really benefit from it.

## Abbreviations

ARAS: Atherosclerotic renal artery stenosis; AUC: Area under the curve; BP: Blood pressure; KDOQI: Kidney Disease Outcomes Quality Initiative; RI: Renal resistive index; MDRD: Modification of Diet in Renal Disease; eGFR: Estimated glomerular filtration rate; ROC: Receiver operating characteristic.

## Competing interests

The authors declare that they have no competing interests.

## Authors’ contributions

RMB and ED and performed statistical analysis and drafted the manuscript; ST, SP, LG, conceived the study and participated in its design; MS performed ultrasound exams; MS, LV, CR, IDP, ED collected data and were involved in the clinical follow-up; DV, SP and LOL participated in design and coordination of the study and critically revised the manuscript. All authors read and approved the final manuscript.
